# Receptor-Independent Interaction of Bacterial Lipopolysaccharide with Lipid and Lymphocyte Membranes; the Role of Cholesterol

**DOI:** 10.1371/journal.pone.0038677

**Published:** 2012-06-07

**Authors:** Filip Ciesielski, Benjamin Davis, Michael Rittig, Boyan B. Bonev, Paul O'Shea

**Affiliations:** 1 School of Biomedical Sciences, University of Nottingham, Nottingham, United Kingdom; 2 School of Biology, University of Nottingham, Nottingham, United Kingdom; Nagoya University, Japan

## Abstract

Lipopolysaccharide (LPS) is a major constituent of bacterial outer membranes where it makes up the bulk of the outer leaflet and plays a key role as determinant of bacterial interactions with the host. Membrane-free LPS is known to activate T-lymphocytes through interactions with Toll-like receptor 4 *via* multiprotein complexes. In the present study, we investigate the role of cholesterol and membrane heterogeneities as facilitators of receptor-independent LPS binding and insertion, which underpin bacterial interactions with the host in symbiosis, pathogenesis and cell invasion. We use fluorescence spectroscopy to investigate the interactions of membrane-free LPS from intestinal Gram-negative organisms with cholesterol-containing model membranes and with T-lymphocytes. LPS preparations from *Klebsiella pneumoniae* and *Salmonella enterica* were found to bind preferentially to mixed lipid membranes by comparison to pure PC bilayers. The same was observed for LPS from the symbiote *Escherichia coli* but with an order of magnitude higher dissociation constant. Insertion of LPS into model membranes confirmed the preference for sphimgomyelin/cholesterol-containing systems. LPS insertion into Jurkat T-lymphocyte membranes reveals that they have a significantly greater LPS-binding capacity by comparison to methyl-β-cyclodextrin cholesterol-depleted lymphocyte membranes, albeit at slightly lower binding rates.

## Introduction

Gram-negative bacteria, co-evolving alongside human hosts, have adapted to occupying available ecological niches as extracellular symbiotes, facultative or true intracellular pathogens. Bacterial interactions with the host reflect their role in the niche, allowing establishment of stable populations or host colonisation relying on evasion of the host immune system by immunomimicry [1], [2], epithelial disruption and invasion of host immune cells. The host response to environmental stimuli, associated with bacterial presence, is governed by cell surface receptor-activated cascades [3], [4]. In addition, an important role in signalling has been attributed to phase heterogeneities in the host cell membranes, such as lipid microdomains or rafts [5], [6]. Some true pathogens directly utilise lateral phase heterogeneities in host plasma membranes to invade host macrophages whilst silencing TLR-mediated inflammatory response [7], [8].

Lipopolysaccharide is the principal component of bacterial outer membranes and its chemical composition is highly species-specific. LPS is released as endotoxin in oligomeric and monomeric form during outer membrane renewal in Gram-negatives and plays an important role in pathogen-host signalling in activating immune response to bacterial presence through Toll-like receptor 4, TLR4 [9], [10]. Receptor activation by LPS is indirect and involves a number of other proteins including LPS-binding protein (LPB) [11], CD14 and MD-2. LPB facilitates LPS binding to the GPI-anchored receptor CD14 [12], which, in turn, stimulates TLR4 dimerisation and initiation of the cellular signalling cascade [13]. Independently of CD14, LPS can also bind to MD-2 either in solution or in association with TLR4 [14]. Evolutionary adaptations in some bacteria have yielded modified LPS with a reduced ability to activate TLR4-mediated pro-inflammatory cascades. *S. typhimurium*, for example, produces LPS with an altered membrane-associated domain, lipid A, which reduces TNF-α expression by monocytes [15].

Mixed lipid membranes containing phosphatidylcholine (PC), sphingomyelin (SM) and cholesterol have been shown to undergo lateral phase separation over a certain compositional and temperature ranges [16] into more ordered, detergent-resistant membrane (DRM) sphingomyelin/cholesterol-rich membrane domains or rafts and phosphatidylcholine-rich disordered membrane phase. Lateral mobility of molecules in the DRM phase is significantly lower than in the PC-rich phase [17]. In cell membranes, this has been used to show that on LPS activation TLR4 partitions, at least transiently, into the less mobile phase [5]. Detergent extraction of DRM domains from cells treated with LPS has revealed association of the TLR4/CD14/MAPK signalling complexes with lipid rafts [18]. Additional role of lipid rafts as mediators of host invasion by *Brucella abortis* has been suggested after observation of class A scavenger receptor co-localisation with lipid rafts during SR-A-mediated internalisation of the pathogen [8].

Besides receptor-mediated association with cellular surfaces, LPS has been shown to interact with membranes of pure PC and of CP/SM/cholesterol mixtures directly from solution [19]. We hypothesize that such direct LPS interaction with membranes is important to facultative pathogens for host invasion in avoidance of triggering immune response but confers no particular advantage to symbiotes. Here, we report results from fluorescence spectroscopic analysis of binding and incorporation of smooth type LPS from a symbiote normally present in the intestinal microflora, *Escherichia coli*, and from facultative pathogens, *Salmonella enterica* and *Klebsiella pneumoniae*, with model membranes and with immortalised human lymphocyte lines. The interaction of each type of LPS with PC/SM/cholesterol membranes is compared to its interaction with pure PC membranes in a quantitative way to assay the role of membrane composition and lateral heterogeneity on LPS/membrane interactions. The role of membrane cholesterol in LPS binding to lymphocyte membranes is also investigated in Jurkat cells before and after treatment with methyl-β-cyclodextrin, MβCD.

The interactions of LPS with each of the membrane types were characterised using a novel fluorescence technique developed in our laboratories. The technique takes advantage of the charge on molecules that on binding and or insertion into membranes leads to small changes of the membrane electrostatic surface potential (see *e.g.* [20]). This leads to a change of the pK of a membrane surface located fluorescence acid-base indicator moiety that at constant pH is observed as changes of the fluorescence due to the binding/insertion interactions. One virtue of the technique is that it can be implemented with both model and living cell membranes [21]. We also utilised a second and complementary fluorescence technique that measures an important membrane quantity known as the dipole potential. Our laboratories pioneered a fluorescent technique to measure membrane interactions that change as the result of changes of the membrane dipole potential. The advantage of using this approach is that this technique illuminates particularly the macromolecular insertion into the body of a membrane [22].

## Results

### The interaction of LPS with artificial lipid membranes

**Figure 1:**
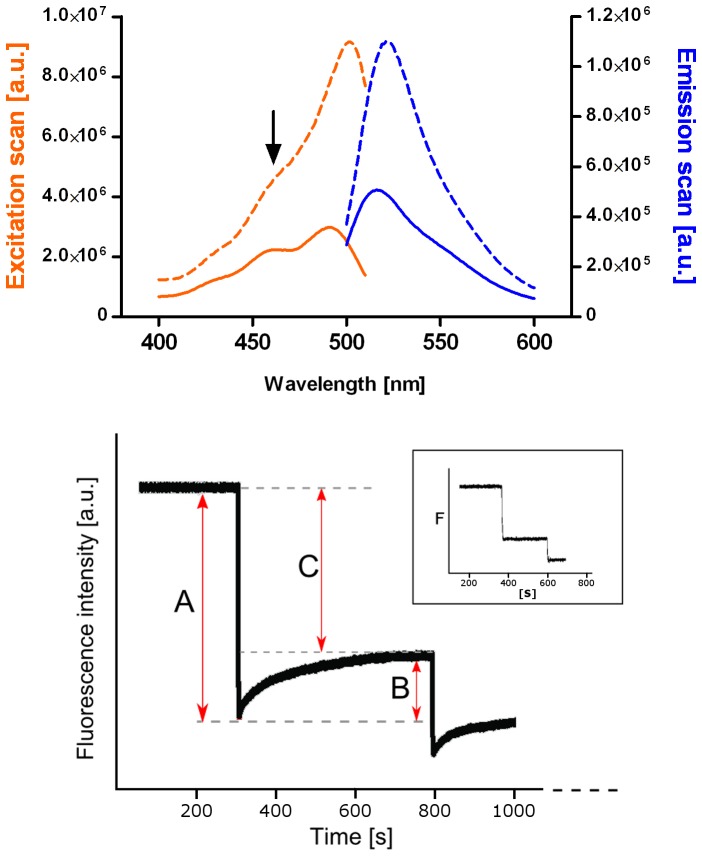
Excitation-emission fluorescence spectra of FPE-labelled phospholipid vesicles (top) before (dashed line) and after (solid line) LPS titration. Arrow indicates a small peak from residual, free FPE in solution; lower panel represents part of the LPS titration curve recorded over time for FPE-labelled PC_55_SM_15_Chol_30_ phospholipid vesicles – initial drop, A, is followed by signal re-equilibration, B. Binding curves (Figure 2) are obtained from measuring changes between the initial signal and the equilibrium state, C. Inset shows titration curve measured for pure PC_100_ vesicles, with significantly smaller difference between the initial binding and re-equilibration stages.

LPS has been shown to insert spontaneously into lipid bilayers and can lead to membrane breakdown at high concentrations [19]. To investigate the lipid specificity of LPS/membrane interactions and obtain quantitative measurements of the binding capacity of membranes for LPS, membranes of different composition were prepared with fluorescein phosphatidylethanolamine (FPE) at levels known not to have any influence on membrane interactions. LPS was added from aqueous solution to large unilamellar vesicle suspensions and fluorescence spectra were recorded. The integrity of FPE-labelled vesicles was assessed by comparing excitation-emission spectra acquired before and after LPS addition (Figure 1a). No major changes in spectral line shape were observed, upon the LPS addition. Changes of the net fluorescence were observed to take place due to the molecular binding reactions and are in accordance with the established mode of action of the FPE reporting system [20], [23]. As the there are no concomitant or slower changes of the spectrum however this indicates that the molecular environment of the FPE is not changed and so the membrane structure is not modified by the interaction of FPE. This indicates that LPS does not disrupt the liposomal membranes over the concentration ranges employed in this study.

**Figure 2:**
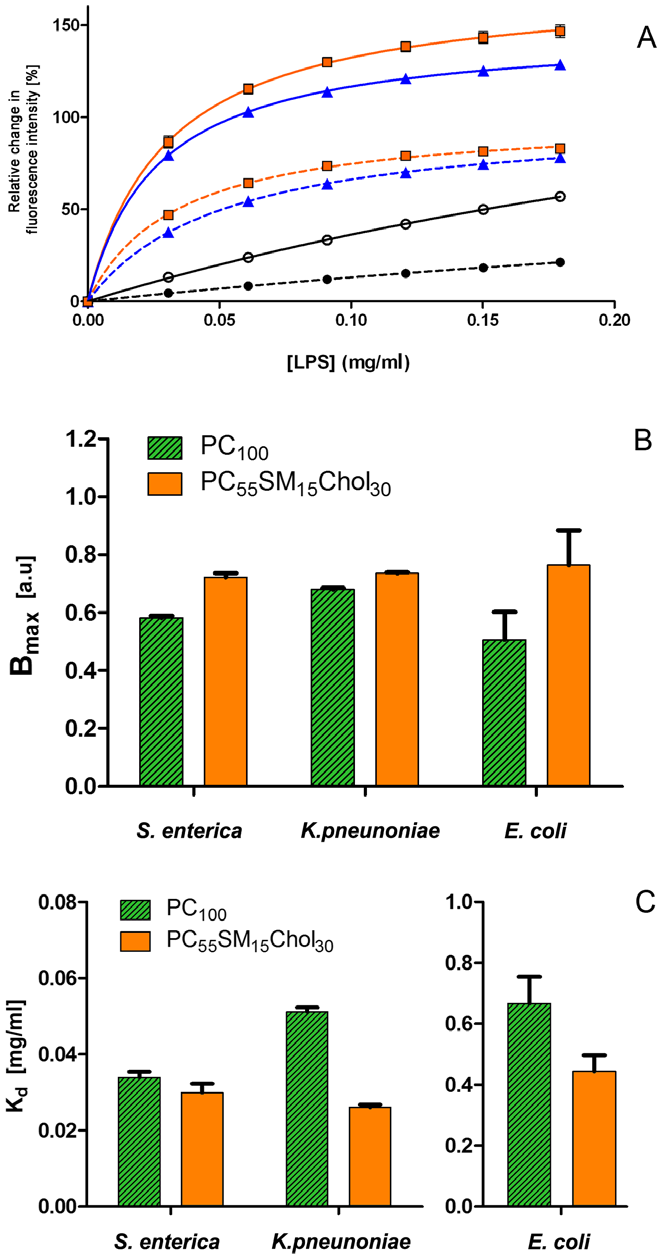
Binding isotherms showing changes in initial fluorescence intensity (*cf.* **Figure 1**) for three types of smooth LPS from *S. enterica*, (squares) *K. pneumoniae* (triangles) and *E. coli* (circles) upon binding to FPE-labelled PC_100_ (dashed lines) or PC_55_SM_15_Chol_30_ (solid lines) phospholipid vesicles. (A). In each case, the average of three repeats was used and the values are shown in comparisons to the data obtained for studies with PC_100_. Histograms of B*_max_* and K*_d_* values, corresponding to (A) but obtained from non-normalized data, are shown in panels (B) and (C), respectively and summarised in Table 1. The values of K*_d_* and B*_max_* for *E. coli* LPS are approximated from the fits, as K*_d_* is greater than the concentration range investigated experimentally. Tolerances in K*_d_* and B*_max_* arise from fitting the data to Equation 1, while error bars in (A) show variance between runs.

All types of LPS carry net negative charges. Addition of LPS to the model membrane preparations resulted in a decrease in fluorescence (Figure 1, lower panel), which is indicative of binding of negative charges to the lipid membranes as described by Wall et al. [24]. Three types of endotoxin (*S. enterica*, *K. pneumoniae* and *E. coli*) were titrated separately against membranes of pure phosphatidylcholine, PC_100_, and mixed membranes of PC, SM and cholesterol: PC_55_SM_15_Chol_30_. This composition was chosen to approximate that of natural cell membranes and also model showing lateral phase properties at the temperature of our studies [25]. Each type of LPS was added cumulatively and the observed fluorescence increase diminishes at each addition as LPS accumulates on the membrane surface until saturation at its binding capacity, B_max_, as shown in Fig. 2A.

The data were, also, fitted to a cooperative binding model (*i.e.* a sigmoidal function) and an F-test was carried out identify if a cooperative binding profile could best define the signal changes. The B_max_ (prior to normalization) and K_d_ values were obtained from the graphs and are presented in Figure 2B,C and numerically in Table 1. For all three types of LPS, B_max_ is higher for endotoxin interactions with mixed lipid bilayer than with the plain PC_100_ (Figure 2B). Assuming all types of vesicles were of uniform size and have the same overall surface area available for LPS binding, the results suggest that more negative charge from LPS can be accommodated on the membrane surface in the presence of lipid domains. In addition, K*_d_* values are observed to be smaller for LPS interactions with the PC_55_SM_15_Chol_30_ membranes (Figure 2c) for all three types of LPS, which indicates that LPS preferentially binds to phase separated mixed lipid membranes. In other words these data indicate that LPS exhibits a preference for membrane microdomain structures within the fluid mosaic lipid membrane. We have previously measured any preferential localisation of FPE between simple fluid-phase PC membranes and those which also contain cholesterol-rich microdomains [25]. Under the experimental conditions of the present study, however, no such preferential localisation takes place. In any event this would only have a bearing on our estimations of the relative binding capacity and not the K*_d_* which are independent of the total fluorescence signals.

**Table 1:** Values of B_max_ and K*_d_* obtained from fluorescence changes recorded in FPE-labelled PC_100_ and PC_55_SM_15_Chol_30_ phospholipid vesicles incubated with LPS of different bacterial origin: *S. enterica*, *K. pneumoniae* and *E. coli*.

LPS source:	PC_100_		PC_55_SM_15_Chol_30_	
	B_max_ [a.u.]	K_d_ [µg/ml]	B_max_ [a.u.]	K_d_ [µg/ml]
*S. enterica*	0.583±0.007	33.93±1.50	0.724±0.014	29.92±2.35
*K. pneumoniae*	0.680±0.005	55.11±1.20	0.735±0.004	26.10±0.65
*E. coli*	0.507±0.096	666.6±152.6	0.764±0.120	444.10±91.14

The measured values of B_max_ are similar for all LPS types, thus indicating that the total number of ‘binding’ sites for the macromolecule are the same for each membrane type (perhaps expected as the total surface area of each membrane system utilised is the approximately the same). On the other hand, the membrane affinity (*i.e.* as K_d_), measured for the *E. coli* LPS membrane interactions, is significantly higher than obtained from the *S. enterica* and *K. pneumoniae* studies. These observations indicate that there is a lower membrane affinity of LPS from the symbiote *E. coli* for either type of membrane when compared to LPS from the opportunistic pathogens.

Another difference between LPS binding to PC_100_ and PC_55_SM_15_Chol_30_ membranes is that the interaction profile for the latter appears to exhibit a greater complexity as shown in Figure 1, lower panel) than PC_100_ (small inset in Figure 1, lower panel). Two distinct stages can be resolved, first a fast initial binding of the negatively charged LPS (labelled A in the figure) is followed by a slower change, B. This latter excursion phase is equivalent to an apparent (slower) recovery of electropositive membrane surface potential. As no positive chares were added the simplest explanation is that it represents the ‘loss’ of negative charge from the immediate membrane surface. It is possible to separate these two phases kinetically by fitting simple rate equations to the time evolution of the signal changes that yield the extent of the signal changes associated with each concentration of added LPS. Thus the B*_max_* and K*_d_* values can be determined separately for each phase as in the simpler case with the PC_100_ membrane system. There is a number of explanations for this phenomenon, such as membrane insertion of some of the charged regions of the macromolecule (as described by [26]) or that the macromolecule located on the membrane surface is undergoing a structural re-arrangement, such that charges move nearer or farther away from the membrane surface and as such exert a greater or lesser effect on the membrane surface potential [23]. Such a rearrangement on the membrane surface appears to have a timescale of minutes, during which a portion of the negative charge moves away from the vicinity of the fluorescent reporter at the lipid surface (labelled B). While a similar pattern is observed for pure PC_100_ membranes, the fluorescence intensity rapidly re-equilibrates on the timescale of seconds (inset to Figure 1, lower panel). As lateral diffusion within the membrane is important in the re-equilibration process, one contributing factor to this complexity may be the presence of phase heterogeneity in the mixed lipid membranes and the associated different diffusion coefficients in the ordered and disordered phases [27]. It is unlikely that shape changes of the membrane vesicles play any role in these phenomena as we are using 100 nm monodisperse unilamellar vesicles, which are thermodynamically very stable under our experimental conditions rather than giant vesicles as in Alam et al. [19].

**Figure 3:**
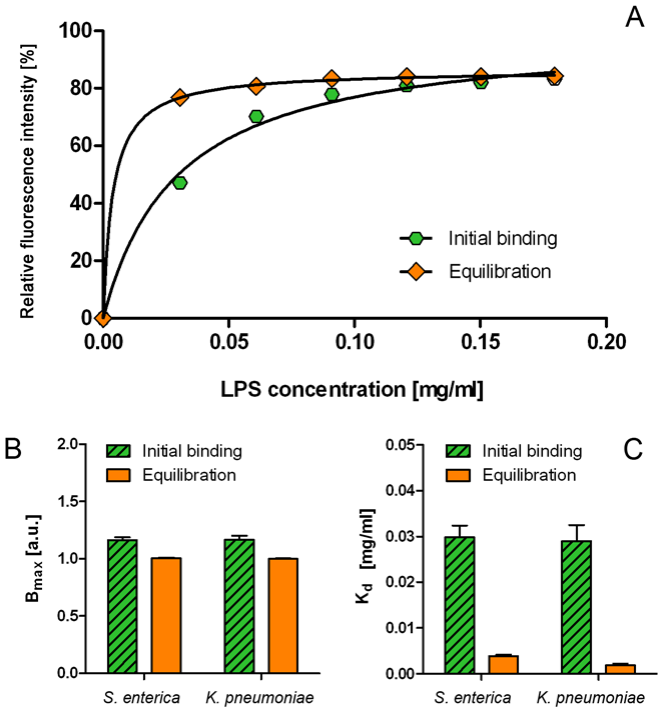
Two stages of *S. enterica* LPS interaction with mixed PC_55_SM_15_Chol_30_ membranes. (A): initial binding (hexagons) and conformational re-equilibration (diamonds). Both datasets were normalized to the starting fluorescent intensities for LPS binding to PC_100_ and the values of B*_max_* are normalized to one for PC_100_ (B) and values for K*_d_* are shown in (C). The average of three repeats is shown and fitted to Equation 1. Similar binding curves were obtained from *K. pneumoniae* LPS binding. The B*_max_* and K*_d_* values for both types of LPS are shown in (B) and (C) and summarised in Table 2.

Receptor-independent binding of LPS to membranes is important to opportunistic pathogens as a route to host invasion. To gain a better insight into this process, the equilibrium properties of initial binding and fluorescence re-equilibration following LPS/membrane interactions were analysed for smooth type LPS from *S. enterica* and *K. pneumoniae*. The binding/equilibration curves are shown in Figure 3A and the corresponding B_max_ and K*_d_* values are compared in Figure 3B,C and summarised in Table 2. LPS from *E. coli* showed similar kinetics of binding to both types of membranes and is not included in the following analysis. The values of B_max_ for the binding step are slightly higher than the re-equilibration step in both types of LPS. This suggests that only a fraction of the charges that bind the lipid bilayer is then rearranged in the second step. The K*_d_* values obtained from initial binding curves are significantly higher than from the re-equilibration step, which suggests that hydrophobic interactions play a significant role in LPS redistribution within the membrane. Differences in kinetic constants between *S. enterica* and *K. pneumoniae* LPS are minimal, which points to a common mechanism of host target engagement.

**Table 2:** Values of B_max_ and K*_d_* obtained from the initial binding and charge-rearrangement detected using FPE-labelled PC_55_SM_15_Chol_30_ phospholipid vesicles.

LPS source:	Initial binding		Equilibration	
	B_max_ [a.u.]	K_d_ [µg/ml]	B_max_ [a.u.]	K_d_ [µg/ml]
*S. enterica*	1.163±0.025	29.81±2.56	1.004±0.004	3.84±0.32
*K. pneumonia*	1.166±0.035	28.97±3.52	0.998±0.005	1.88±0.34

### Partial insertion of LPS into phospholipid membranes

**Figure 4:**
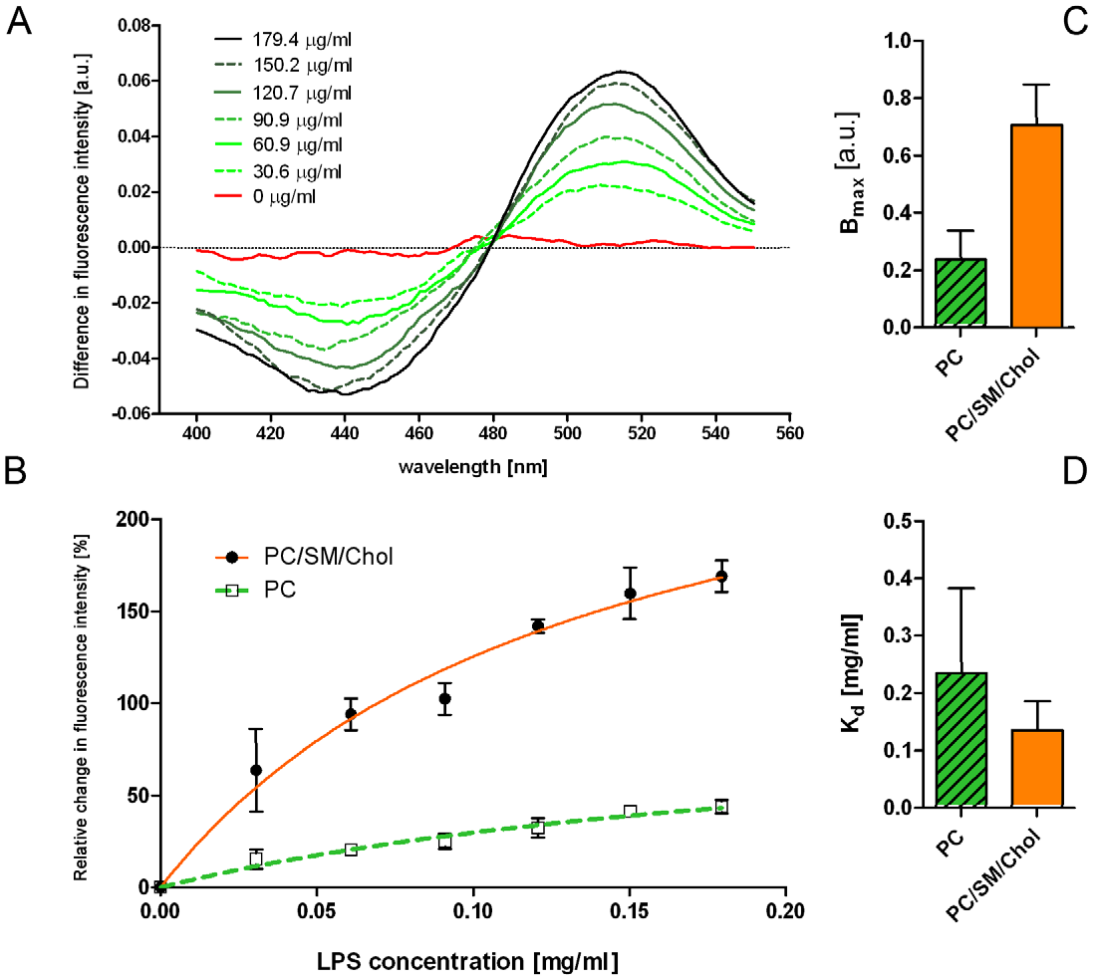
Difference fluorescence spectraof di-8-ANEPPS-labelled PC_100_ and PC_55_SM_15_Chol_30_ phospholipid vesicles before and after titration of LPS from *S. enterica* (A). The addition of endotoxin to both types of vesicles results in red shift. Changes in R460/520 ratio were plotted in (B) and fitted to Equation 1. The graphs are normalized to B*_max_* of the PC_100_ and to the starting fluorescence intensity. The B*_max_* and K*_d_* values were estimated from the fits and are presented in (C) and (D), respectively.

We investigated the role of membrane composition on insertion of LPS into membranes using the fluorescent probe di-8-ANEPPS (4-[2-[6-(Dioctylamino)-2-naphthalenyl] ethenyl]-1-(3-sulfopropyl)-pyridinium, inner salt), one advantage of this probe is that it can yield information about the penetration of macromolecules into the membrane interior [22]. Thus by measuring the emission at different excitations, the ratio (R_460/520_) has been shown to be a good approximation of the level of the membrane dipole potential and any concomitant changes due to molecular interactions. Smooth LPS from *S. enterica* was titrated into di-8-ANEPPS-containing phospholipid vesicles made up of PC_100_ and PC_55_SM_15_Chol_30_. Excitation was measured before and after endotoxin addition and subtracted to obtain from the difference spectra a red shift in fluorescence (Figure 4A). The magnitude of such shifts has been shown to be dependent on the concentration of the interacting species ([28]), thus as more LPS became bound to the membrane this led to greater changes of the membrane dipole potential with one interpretation being that the LPS penetrates the membrane interior. R_460*/*520_ ratios were measured before and after LPS addition and differences were plotted against LPS concentration (Figure 4B). The experiments were carried out with pure lipid, PC_100_, and mixed lipid membranes, PC_55_SM_15_Chol_30_, to investigate the role of lateral phase separation and the presence of cholesterol. Comparison between the membrane binding of smooth LPS from opportunistic pathogens *S. enterica* and *K. pneumoniae* showed very similar kinetics thus we report only our studies of membrane insertion of *S. enterica* LPS.

Difference spectra and kinetic constants of smooth *S. enterica* LPS binding to PC and to mixed lipid membranes are shown in Figure 4 and summarised in Table 3. The binding capacity (*i.e.* as B_max_) for LPS is greater in PC_55_SM_15_Chol_30_ membranes compared to PC_100_. The K*_d_* from LPS binding to pure PC_100_ was found to be larger than that found for the mixed bilayer. Combined, the FPE and di-8-ANEPPS data reveal a preference for LPS from all species to insert into membranes that would exhibit microdomains *i.e.* most likely as laterally segregated cholesterol-containing triple mixtures over the pure PC.

**Table 3:** Values of B_max_ and K*_d_* obtained from fluorescence changes recorded from di-8-ANEPPS-labelled PC_100_ and PC_55_SM_15_Chol_30_ phospholipid incubated with *Salmonella enterica* LPS.

LPS source:	PC_100_		PC_55_SM_15_Chol_30_	
	B_max_ [a.u.]	K_d_ [µg/ml]	B_max_ [a.u.]	K_d_ [µg/ml]
*S. enterica*	0.239±0.098	236±146	0.708±0.139	136±50

### The interactions of LPS with T Lymphocytes

The role of cholesterol in binding of LPS from *S. enterica* to membranes of live cell and the putative role of lipid rafts were investigated in Jurkat cells, labelled with FPE. The lipid composition of cell membranes is approximated by the triple lipid mixture model preparation used in this study (PC_55_SM_15_Chol_30_ system), which are known to undergo phase separation into raft-like domains (akin to cell membranes [25]). For comparison, treatment with MβCD depletes membranes of cholesterol and reduces membrane propensity to phase separate into raft-like lateral microdomains [28].

**Figure 5:**
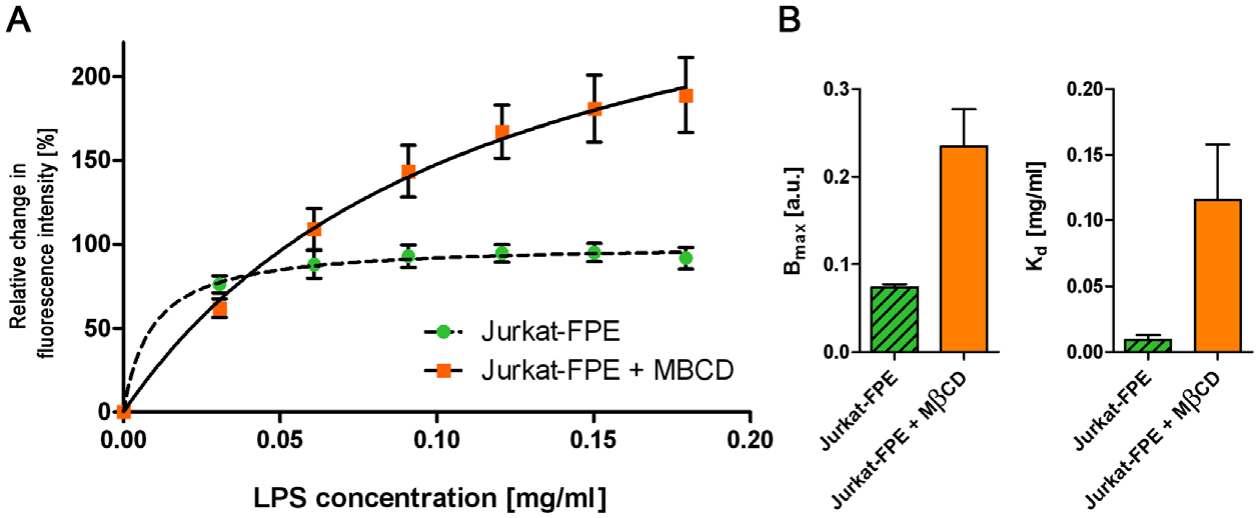
Binding isotherms of LPS from *S. enterica* to FPE-labelled Jurkat cells and to Jurkat cells, from which cholesterol has been removed with M*β*CD prior to addition of LPS (A). Both curves are normalized to initial fluorescence intensity of untreated cells. Values for B*_max_* and K*_d_* values are show in panel (B).

LPS from *S. enterica* was titrated into a suspension of T lymphocytes and the membrane interactions monitored as changes in FPE fluorescence. Studies were also performed with Lymphocytes which had been pre-treated with MβCD to deplete the cell membrane cholesterol. Fluorescence intensity is shown in Figure 5 along with B_max_ and K_d_. Experimental scatter in the LPS titration curves hindered differentiation between binding and charge re-arrangement and so only the composite total signal profiles can be reported (*i.e.* comprising both the binding and rearrangement phases of the LPS interaction – defined in Figure 1). The observed LPS binding capacity B_max_ values are higher in MβCD-treated Jurkat cells, suggesting a greater LPS binding capacity to cholesterol-depleted membranes. By contrast, the corresponding dissociation constant is also higher, which shows a lower affinity for LPS after cholesterol depletion by comparison to untreated cells. The apparent increased LPS binding affinity observed for the MβCD treated cells is interesting and may be the result of combining the 2 interaction phases we define in Fig. 1. Alternatively (or in addition) it may reside in the possibility that MβCD is only known to remove cholesterol whereas in cells microdomains may be stabilised by several other factors in addition to cholesterol. These factors may include cytoskeletal elements, ECM and other lipid components unaffected by MβCD treatment or modulations of the levels of the fluid membrane free volume by cholesterol [29]. The lower K*_d_* values observed in untreated cells is consistent with our FPE fluorescence results from mixed lipid PC_55_SM_15_Chol_30_ membranes (see earlier section) and supports the hypothesis of a greater LPS affinity for phase separated/lipid raft-containing membranes.

### LPS insertion into Lymphocyte cell membranes

**Figure 6:**
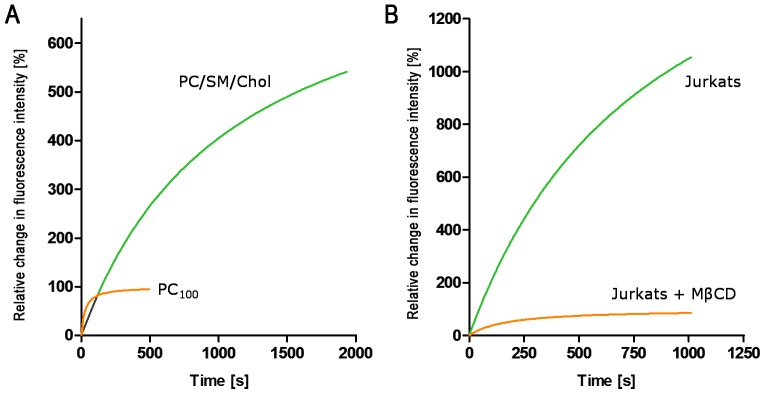
Time dependent changes in fluorescence from di-8-ANEPPS-labelled phospholipid vesicles and from Jurkat cells exposed to a single concentration of *S. enterica* LPS. Panel (A) shows changes in di-8-ANEPPS fluorescence recorded over time from phospholipid vesicles and panel (B), form Jurkat cells; orange lines show membranes without lipid domains (either PC_100_ or M*β*CD-treated Jurkat cells). The PC_55_SM_15_Chol_30_ curve is normalised to PC_100_ and Jurkats fluorescence is normalised to M*β*CD-treated cells.

The time-dependent insertion of LPS from *S. enterica* into the Jurkat lymphocyte cell membranes and into the cholesterol-depleted membranes (MβCD-treated) was investigated following changes in the fluorescence of membrane embedded di-8-ANEPPS and was compared to model systems PC_55_SM_15_Chol_30_ and PC_100_, respectively (Figure 6). Following addition of LPS, significantly longer equilibration times on the order of 30 minutes were required to achieve a fluorescence steady state in the live system by comparison to the model membrane system. Such observations may arise due to structures absent in a model system compared to that of the cellular system. This strategy has been employed previously in other systems and allows some discrimination between purely lipid-based interactions and those that may involve receptor systems (see *e.g.* Asawakarn et al. [28].

LPS insertion into membranes without lipid domains, either PC_100_ or Jurkats treated with MβCD, was notably faster by comparison to PC_55_SM_15_Chol_30_ and untreated cells. The rate constant of LPS insertion into the former is about 40 times higher than for PC_55_SM_15_Chol_30_ phospholipid vesicles and about 4 times greater in the case of MβCD-treated Jurkat cells compared to untreated cells with correspondingly lower K_d_ values (Figure 6). However, the binding capacity for LPS was greater both in cholesterol-containing, native, Jurkat membranes and in PC_55_SM_15_Chol_30_ phospholipid vesicles, where raft-like lipid domains were predicted or present. Qualitatively, these results suggest that by contrast to LPS binding, LPS insertion occurs with higher affinity in pure PC_100_ membranes and in cholesterol-depleted Jurkat cells albeit at lower binding capacity by comparison to PC_55_SM_15_Chol_30_ membranes and native Jurkat cell membranes. These observations are paralleled by slower kinetics of LPS insertion in the presence of cholesterol. This suggests a role of lateral diffusion and lipid order in the organised lipid sub-phases as retardants of LPS reorganisation and insertion into mixed lipid membranes both *in vitro* and *in vivo*.

## Discussion

The role of membrane heterogeneities in host-pathogen interactions has been investigated for receptor-mediated pathogen recognition during host response to bacteria. Key mediators of the LPS response cascade, molecular complexes involving TLR4 [30] and CD14 [18], are recruited to membrane lateral domains following exposure to bacterial LPS. Advantageous to some bacterial pathogens, activation of TLR4-mediated inflammatory cascades is suppressed and the host membrane is engaged away from pro-inflammatory receptor complexes and used in host cell invasion [8], [31]. Here, we used fluorescence spectroscopy to investigate the interactions of LPS from *E. coli*, *S. enterica* and *K. pneumoniae* with model and live cell membranes and the role of cholesterol and lateral phase separation in this interaction.

The interaction between LPS from all three bacterial species showed preference for raft-containing cholesterol-rich membranes, characterised by lower dissociation constant and higher binding capacity. Interestingly, K_d_ for *E. coli* LPS was an order of magnitude higher than the corresponding values for the other *K. pneumoniae* and *S. enterica*. Lower affinity may reflect an evolutionary adaptation of *E. coli*, which is a normal resident of the intestinal microflora. The molecular mechanism behind this is likely to reflect differences in the oligosaccharide, as *E. coli* and *S. enterica* have very similar lipid A moiety, which includes asymmetric 4∶2 acylation of the sugars. By contrast, the lipid A moieties of *S. enterica* and *K. pneumoniae* are asymmetrically 4∶2 and symmetrically 3∶3 acylated (for review see [32]), yet the two types of LPS show very similar K_d_ values. The overall similarity of the lipid A moieties of the three types of LPS investigate, all hexa-acylated, is the likely reason for the similarity in B_max_. Yet, subtle differences in acylation between *E. coli* and *S. enterica* on the one hand and *K. pneumoniae*, on the other, may account for the marginally greater difference in B_max_ in the former two by comparison to the latter. The asymmetric acylation of the former two may provide a less ordered acyl chain region near the di-acyl moiety and a putative binding site for cholesterol.

Lipid reporters with an acid-base surface-localised fluorophore, such as FPE, is sensitive to changes in the electrostatic environment within the membrane lipid headgroup region and can detect charge association with the membrane surface, as well as movement of charges due to lateral redistribution or following insertion of charged species into the membrane interior [24]. Dissociation constants, determined from initial changes in FPE fluorescence are markedly higher for all LPS types by comparison to K_d_ values determined after equilibration. This is likely to reflect a multistep process, during which LPS oligomers associate with the membrane where conversion to monomeric form of LPS leads to final insertion into the membrane. Such process is likely to be lateral diffusion-limited and to require loner equilibration times in raft-containing membranes, where lipid lateral diffusion rates are lower than in pure PC membranes [17]. Indeed, analysis of time-dependent changes in di-8-ANEPPS fluorescence during LPS insertion (Figure 6) shows higher rates of insertion into PC membranes and cholesterol-depleted cells by comparison to PC_55_SM_15_Chol_30_ and native Jurkat membranes, respectively. However, the corresponding membrane insertion capacities in the absence of cholesterol are lower both in the model and in the cell systems, which is likely to reflect optimal packing of the lipid A acyl moieties within ordered, cholesterol-rich membrane domains in these systems.

The latter observation has a profound implication on our understanding of LPS interactions with cell membranes. Experimental evidence points to transient re-localisation of LPS-activated TLR4 receptor complexes into lipid rafts during initiation of immune response [30], which implies steady state localisation of inactive TLR4 in the fluid membrane sub-phase. By contrast, immunosilent host invasion, during which TLR4 cascades remain silent, involves a direct interaction between invading bacteria and lipid raft within the host membrane [7]. Results in the present study suggest preferential insertion of LPS into membrane rafts, which may be an important part of or contribute to immunosilent host invasion by pathogenic bacteria.

In summary, fluorescence spectroscopy was used to investigate the interactions between LPS from *E. coli*, *S. enterica* and *K. pneumoniae* with lipid membranes composed of PC alone or containing PC, sphimgomyelin and cholesterol. The role of cholesterol on LPS binding was also investigated in Jurkat cells or in cholesterol-depleted MβCD-treated jurkat cells. Biding of all types of LPS to model membranes was characterised by lower dissociation constants and similar or slightly higher capacity in the triple mixtures. The K_d_ values determined for LPS from non-pathogenic *E. coli* were significantly higher than the corresponding values from *S. enterica* and *K. pneumoniae*. LPS insertion showed preference for triple-lipid membranes with little difference between species of origin. Native Jurkat membranes showed higher binding capacity for LPS by comparison to cholesterol-depleted cells, although with correspondingly higher K_d_ values. LPS insertion into model membranes and cells showed slower kinetics, which correlates with slower lateral diffusion and suggests preference of LPS insertion for ordered, cholesterol-rich membrane domains. Therefore, association of previously reported LPS-induced activation complex with lipid rafts may follow or be coincidental with incorporation of LPS into cholesterol-rich lateral domains.

## Materials and Methods

### Liposome preparation

Phosphatidylcholine bilayers (PC_100_) were prepared using egg lecithin mixture (Lipid Products, UK) and mixed lipid bilayer (PC_55_SM_15_Chol_30_) with detergent-resistant domains was prepared from phosphatidylcholine, sphingomyelin (SM) and cholesterol (Chol) at the molar ratio of 55∶15∶30. Sphingomyelin and cholesterol were supplied from Sigma Aldrich (Poole, UK) and used without further purification. Phospholipid vesicles were prepared as described previously [33]; briefly desired volumes of lipid in chloroform∶methanol (solvent ratio 5∶1) were measured before the solvent was evaporated under a stream of oxygen free N_2_ gas. The resulting lipid film was resuspended in LPS buffer (10 mM Tris, 1 mM EDTA and pH 7.4) before freeze-thawing 10 times with liquid N_2_ and hot (50°C) water bath. The resulting multilamellar vesicles (MLVs) were extruded through polycarbonate filter of pore-size 100 µm (Nucleophore Filtration Products, USA) using N_2_ gas and pressure extruder (Lipex Biomembranes Inc., Vancouver, Canada) to generate 13 mM unilamellar lipid vesicle suspensions. Lipopolysaccharides were purchased from Sigma, UK; and used without further purifications. LPS samples were prepared by suspending LPS powder in LPS buffer (10 mM Tris, 1 mM EDTA and pH 7.4) to a final concentration of 6.67 mg/ml.

### Cell culture

Jurkat T-lymphocytes (E6-1 clone) were obtained from the European Collection of cell cultures (ECACC) and cultured in 90% RPMI 1640 medium with 10% heat inactivated foetal calf serum (FCS), L-glutamine (100 µm/ml) and Penstrep (Penicillin and streptomycin mixture, 100 µm/ml). Culture medium was replaced every 3 days and the colony was maintained at 37°C and 5% CO_2_ with a cell density between 1×10^5^ cells/ml and 1×10^6^ cells/ml.

### Fluorescent Labelling

Fluoresceinphosphotidylethanloamine (FPE) was synthesised according to the published methods of Wall et al [24]. Phospholipid vesicles were labelled with either FPE or di-8-ANEPPS (Molecular Probes, Leiden, The Netherlands) to a final concentration of 0.2% (molar) and in the presence of ethanol (1.5% (v/v)) by adding the fluorescent dye as an ethanol solution to the vesicles stock and incubating at 37°C for 1 h for FPE and 1.5 h for di-8-ANNEPS (Asawakarn 2001). In order to remove unbound probe, FPE-labelled vesicles were additionally filtered through a PD-10 column equilibrated with buffer (10 mM Tris, 1 mM EDTA and pH 7.4). As di-8-ANEPPs has a low quantum yield in water relative to a lipid environment, this step was not required for di-8-ANEPPs labelled vesicles.

To label lymphocytes with FPE confluent cell cultures (1×10^6^ cells/ml) were harvested by gentle centrifugation (300×g for 5 min at 25°C) and resuspended in sucrose-Tris buffer (280 mM sucrose, 10 mM Tris, 1 mM EDTA and pH 7.4) to a concentration of 1×10^6^ cells/ml. FPE solution was added to a final concentration of 8.8 nmoles of FPE per 1×10^6^ cells and incubated for 1 h at 37°C.

To label Lymphocytes with di-8-ANEPPS confluent cell cultures (1×10^6^ cells/ml) were harvested by gentle centrifugation (300×g for 5 min at 25°C) and resuspended in sucrose-Tris buffer (280 mM sucrose, 10 mM Tris, 1 mM EDTA, pH 7.4) to a concentration of 0.5×10^6^ cells/ml. Di-8-ANEPPS was added to a concentration of 6 nmoles per 1×10^6^ cells) and the sample was incubated for 1.5 h at 37°C.

Cholesterol-depleted, fluorescently-labelled (either FPE or di-8-ANEPPS) T-lymphocytes were prepared using the aforementioned labelling protocols before briefly exposing the cells to MβCD solution (66 mg/ml) for 4 min. Cells were then harvested by gentle centrifugation (300×g for 5 min at 25°C) and returned to sucrose-Tris buffer. This MβCD treatment protocol is adapted from the method of [34].

### FPE fluorescence measurements

FPE-experiments were conducted using labelled phospholipid vesicles at a concentration of 390 µM of total lipid and excitation scan (450–520 nm range, emission measured at 530 nm) followed by emission scan (measured emission over 520–590 nm range with excitation at 490 nm) were recorded at 37°C before and after LPS additions to inspect vesicle labelling efficacy. LPS was added cumulatively to a final concentration of 24 mg per µmole of total lipids and fluorescence was measured with a continuous excitation at 490 nm and emission detection at 520 nm. Changes in fluorescence were plotted as the inverse of the percentage change in signal and fitted to the hyperbolic equation (equation 1).
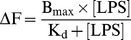
(1)The FPE-experiments on labelled lymphocytes were carried at a concentration of 4×10^4^ cells/ml and LPS was added in 6 steps to a final concentration of 12 mg per 1×10^4^ cells. Data analysis was conducted as described previously for artificial membranes [23].

### Di-8-ANEPPS fluorescence measurements

Di-8-ANEPPS-labelled PC_100_ and PC_55_SM_15_Chol_30_ phospholipid vesicles were used at a concentration of 0.41 µmole of total lipid per ml and excitation scans were recorded at 37°C (400–550 nm range, emission measured at 590 nm). Fluorescence measurements were taken at 590 nm upon excitations at 460 and 520 nm (denoted F_460/590_ and F_520/590_ respectively). The ratio 460 nm/520 nm (R_460/520_) was then calculated using the following equation:
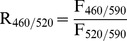
(2)LPS from *S. enterica* was then added to six individual vesicle samples of both compositions (PC_100_ and PC_55_SM_15_Chol_30_) at a linear concentration range between 76 µg and 444 µg of LPS per 1 µmole of total lipid. Liposome samples were incubated with different concentrations of LPS for 25 min at 37°C before excitation scans and R_460/520_ ratio were recorded. Excitation spectra were normalized before subtracting spectra in the presence of each concentration of LPS from those in the absence of the molecule in order to give difference spectra. In addition, changes in the R_460/520_ ratio on addition of LPS were plotted against LPS concentration and fitted to a hyperbolic equation [22].

### Time evolution of Di-8-ANEPPS fluorescence changes

Di-8-ANEPPS-labelled PC_100_ and PC_55_SM_15_Chol_30_ phospholipid vesicles were used at a concentration of 0.41 µmole of total lipid per ml and a single concentration of LPS (0.3 mg LPS per 1 µmole of total lipid) was added to each membrane and R_460/520_ ratio was measured as a function of time for approximately 25 min.

Di-8-ANEPPS-labelled T-lymphocytes with and without MβCD pre-treatment were used at a concentration of 2×10^4^ cells/ml in sucrose-Tris buffer and a single concentration of LPS was added to each and R_460/520_ ratio was measured as a function of time for approximately 15 min.
